# Functional Neuroimaging of Human Hypothalamus in Socioemotional Behavior: A Systematic Review

**DOI:** 10.3390/brainsci12060707

**Published:** 2022-05-30

**Authors:** Andrea Caria, Ginevra Matilde Dall’Ò

**Affiliations:** Department of Psychology and Cognitive Science, University of Trento, 38068 Rovereto, Italy; ginevramatilde.dallo@studenti.unitn.it

**Keywords:** hypothalamus, social brain network, prosocial, antisocial, emotion, oxytocin, affiliative, parent-child, love, partner, cooperation, trust, altruism, aggressive, defense, fMRI, neuroimaging

## Abstract

There exist extensive animal research and lesion studies in humans demonstrating a tight association between the hypothalamus and socioemotional behavior. However, human neuroimaging literature in this direction is still rather limited. In order to reexamine the functional role of this region in regulating human social behavior, we here provided a synthesis of neuroimaging studies showing hypothalamic activation during affiliative, cooperative interactions, and in relation to ticklish laughter and humor. In addition, studies reporting involvement of the hypothalamus during aggressive and antisocial interactions were also considered. Our systematic review revealed a growing number of investigations demonstrating that the evolutionary conserved hypothalamic neural circuity is involved in multiple and diverse aspects of human socioemotional behavior. On the basis of the observed heterogeneity of hypothalamus-mediated socioemotional responses, we concluded that the hypothalamus might play an extended functional role for species survival and preservation, ranging from exploratory and approaching behaviors promoting social interactions to aggressive and avoidance responses protecting and defending the established social bonds.

## 1. Introduction

There exists longstanding evidence of dysfunctions of socioemotional behavior in animals and humans after hypothalamic lesions [1,2,3,4,5]. More recently, optogenetic studies in animals clearly demonstrated that hypothalamic neurons can actively control socioemotional behavior such as fear and defensive responses [6,7,8,9,10]. Furthermore, hypothalamic peptides such as oxytocin and arginine-vasopressin have recently attracted great interest for their implications in typical and atypical socioemotional behavior [11,12,13,14,15,16,17]. A large number of controlled trials demonstrated that exogenous oxytocin or arginine-vasopressin administration can significantly influence human socioemotional responses. Intranasal oxytocin administration was associated with increased exploration of the eye region [18], enhanced facial emotional recognition [19,20], mind reading abilities [21], and with increased trust in healthy individuals [22]. Neuroimaging studies indicated that intranasal oxytocin influences behavior by modulating activity and interaction of several social brain areas [23,24,25,26,27], for instance, by reducing amygdala response to emotional faces [28]. On the other hand, under certain circumstances oxytocin administration has been also shown to induce non-prosocial and aggressive responses in humans [29,30,31]. Remarkably, a growing body of research showed that intranasal oxytocin and vasopressin administration can in some cases ameliorate social abilities in Autism Spectrum Disorders (ASD) [32,33,34,35,36,37,38]. On the basis of these impressive outcomes, a recent review of structural and functional MRI investigations investigating morphological and functional alterations of the hypothalamus in ASD reported two main findings: a reduction of hypothalamic gray matter (GM) volume and a functional hypoactivation of this region during face processing and social interaction, in both adults and children [17].

However, despite the well-documented role of the hypothalamus in supporting and regulating socioemotional responses from quite heterogenous perspectives, our understanding of the anatomical and functional properties of the hypothalamic nuclei in relation to human socioemotional behavior still remains limited. A rather small number of neuroimaging studies have so far directly investigated the role of this region in human socioemotional behavior. Surprisingly, the hypothalamus is also often elusive in several fMRI studies broadly investigating the neural correlates of socioemotional responses. In order to clarify the role of the hypothalamus in typical socioemotional behavior, we here conducted a systematic review of human neuroimaging studies reporting hypothalamic activation in response to several different socioemotional stimuli and contexts. In particular, we examined the involvement of the hypothalamus during affiliative, cooperative, as well as aggressive and antisocial interactions. We also considered studies investigating neural correlates of laughter, in particular ticklish laughter and humor, two highly rewarding behaviors tightly linked to socioemotional interaction and communication [39,40,41]. A concise description and analysis of the selected studies is first provided. Conclusions are then drawn in light of the hypothalamic engagement during both prosocial and antisocial behaviors.

## 2. Methods

**Figure 1:**
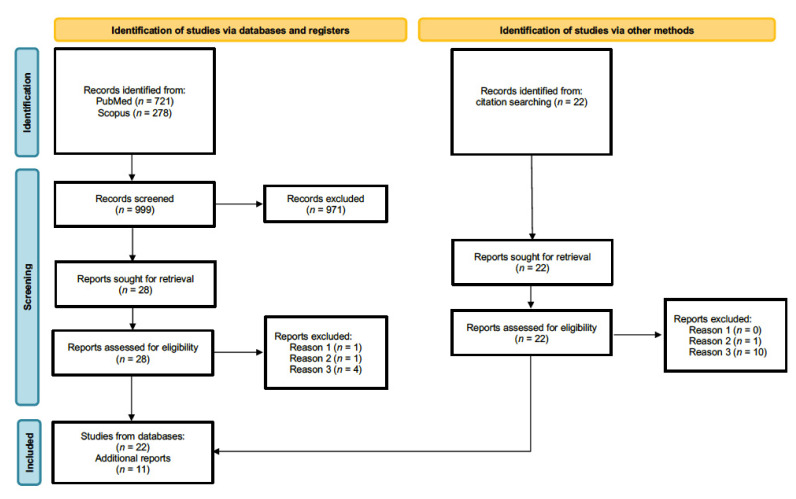
Study flow chart. Reason 1: do not investigate neural basis of maternal love [48]. Reason 2: articles on clinical populations [49,50]. Reason 3: do not report hypothalamic activation.

Our systematic review was conducted in accordance with PRISMA guidelines [42]. We conducted separate searches with Scopus and PubMed databases using the following combination of keywords: hypothalamus AND fMRI AND (emotion OR social), April 2022. In total, 462 and 240 publications resulted from initial PubMed and from Scopus searches, respectively. In addition, further searches aiming to refine and extend our selection process were conducted using the following combination of keywords: hypothalamus AND fMRI AND (affiliative OR trust OR humor OR humor OR tickling OR aggressive OR antisocial OR threat). An additional 259 articles resulted from PubMed and 38 from Scopus. An initial screening allowed us to exclude articles using different neuroimaging modalities, conference proceedings, and articles not available in English. We then selected only articles reporting original research studies and excluded reviews and opinion articles. Consecutively, we excluded articles on animal models, clinical populations, studies investigating only genetic aspects of the hypothalamus or hypothalamic neuropeptides, and those not employing socioemotional stimuli. Application of these exclusion criteria resulted in 28 relevant articles. Additional 22 studies missing from initial searches were identified through cross-references and relevant review articles [43,44,45,46,47]. Full-text inspection of the 50 publications led to a further rejection of three articles based on the above-mentioned criteria. In addition, 14 studies surviving the previous selection process were ultimately discarded because hypothalamic activation was actually not reported. Finally, 33 articles were considered for our review (a flow chart of the literature search procedure is depicted in Figure 1). Studies were grouped on the basis of the type of social behavior as follows: parental interactions, pair bonding, other prosocial interactions, laughter, and social threat.

## 3. Results

**Figure 2:**
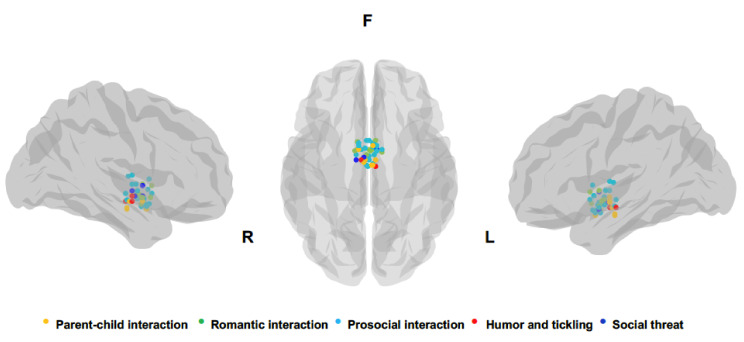
Peak voxel coordinates of hypothalamic clusters reported in the studies included in the review. All peaks were plotted on a brain mesh of Ch2 template using the BrainNet Viewer [51]. On the left and right side are depicted sagittal views and in the center the axial view (bottom-up).

A chronological summary of all studies analyzed, synthetizing socioemotional context, number and sex of participants, main stimuli comparison leading to hypothalamic activity, and coordinates of peak activation of hypothalamic cluster are reported in Table 1. Peaks for each hypothalamic activation reported in the studies analyzed are also depicted in Figure 2. The number of studies identified for each socioemotional context is the following: 8 parental interactions, 7 pair bonding, 11 other prosocial interactions, 3 laughter, and 4 social threat.

### 3.1. Parental Interactions

One of the first fMRI studies reporting hypothalamic activation in relation to mother–infant relationships was conducted by Lorberbaum and colleagues, who explored the brain basis of human maternal behavior [52]. The authors observed that primiparous mothers listening to infant cries, as compared with either noise control sounds or rest conditions, showed increased activity in several cortical and subcortical regions including hypothalamus/mammillary bodies, vicinity of the lateral septal region and amygdala along with the midbrain, dorsal and ventral striatum, medial thalamus, and medial prefrontal and right orbitofrontal cortices [52]. Additional studies investigated the neural mechanisms underlying maternal bonding by assessing brain responses during presentation of infant pictures [54,56]. Observation of mothers’ own infants’ faces as compared with unknown infants’ faces was also associated with increased activity of the hypothalamus and amygdala, and other regions of the dopaminergic reward processing system, regardless of the emotional valence of infant faces (happy or sad) [54,56].

A further study investigating maternal attachment behavior through presentation of videoclips of their own infant showing play or separation scenarios reported significant activations of the right hypothalamus along with midbrain, anterior insula, orbitofrontal and dorsomedial prefrontal cortices, and other regions [55]. A specific activation of the left hypothalamus was measured when mothers viewed their own infant in the play situation as compared with the separation situation, suggesting lateralized effects in relation to rewarding and empathic responses and discomfort experience or maternal response to mitigate child stress.

Interestingly, Strathearn and colleagues [56] showed that hypothalamic activity reflected maternal attachment as measured with the Adult Attachment Interview [79]. Secure mothers displayed a significantly greater activation in the hypothalamus/pituitary regions, the lateral prefrontal cortex bilaterally, and the left medial prefrontal cortex and with respect to insecure/dismissing mothers. They also observed that brain activation in the ventral striatum and in the hypothalamus/pituitary regions positively correlated with mothers’ peripheral oxytocin response to their infant contact at 7 months after delivery, indicating a direct link between the oxytocinergic system and mother–infant attachment [56].

On the other hand, hypothalamic activity was also observed in relation to socioemotional negative experiences. For instance, Ho and colleagues observed increased activity of the right hypothalamus and left amygdala in mothers with greater tendencies to experience personal distress in reaction to unpleasant child response [58]. Though, a positive coupling between the septo-hypothalamic area was associated with less cortisol reactivity during distressed conditions [58], indicating that hypothalamus–septal interactions might mediate down-regulation of stress-related cortisol reactivity [80] so as to appropriately tune parental behavior [81].

A further indication of the hypothalamic role in affiliative experience was provided by Moll and colleagues, showing significant activity in a neural continuum including the preoptic area, the ventromedial hypothalamus, the left septal/preoptic-anterior hypothalamic area, and the medial frontopolar cortex when male and female participants were presented with visual sentences, either with positive or negative valence, describing social scenarios evoking feelings of care and tenderness towards an attachment figure, such as one’s mother, father, or offspring [57]. Notably, no differences between men and women were observed in relation to overall ratings of affiliative feeling and valence of presented stimuli, suggesting a comparable effect also at the neural level. The observed activation of the septal/preoptic-anterior hypothalamic region was stronger for positive emotional valence of affiliative scenarios [57].

Finally, involvement of the hypothalamus was also observed in fathers’ responses to infants [59]. Li and colleagues observed that neural responses of first-time fathers presented with either own’s or others’ infant cry stimuli was highly similar to those of first-time mothers, and included activity in the midbrain thalamo-cingulate circuit, fronto-insular, and dorsomedial prefrontal regions. Increased engagement of the left hypothalamus and dorsal anterior cingulate cortex (ACC) was observed in fathers with more negative emotional reactions to infant cry, further indicating a relationship between maladaptive parental neural responses and stress-related hypothalamic activity.

### 3.2. Pair-Bonding

Maternal and romantic love, because of their analogous evolutionary goal of maintaining and perpetuating the species, and their intrinsic rewarding experience, are posited to be mediated by common brain systems [53,82]. In particular, the hypothalamus was proposed as key region regulating both human adult interpersonal relationship and adult–child interaction. Bartels and Zeki, comparing neural activations in mothers presented with pictures of their own children (maternal love) with those elicited by presentation of pictures of their partners (romantic love) [83], showed several overlapping regions in the dopaminergic reward system, although only romantic love and not maternal love elicited activation in the hypothalamus [53]. This single study did not show hypothalamic activity associated with mother–infant relationship maternal love but several other studies reported its involvement [54,55,56] and corroborated indications of a partially common neural substrate for different types of attachment relationships.

In relation to pair bonding, Acevedo and colleagues (2012) reported activity of the right hypothalamus when participants involved in long-term romantic love viewed facial images of their partner. Such activation was specifically correlated with sexual frequency, possibly indicating the strength of the relationship [84]. More recently, the same authors reported hypothalamic activation in male and female participants that were requested to recall romantic events when presented with happy or sad pictures of their spouse, both in a first measurement session at around the time of the wedding and in a second session after one year [63]. In addition, these authors observed an interaction effect between a genetic marker of social and empathic abilities (OXTR rs53576—G alleles, [85,86,87]) and altruism scores in the left and right hypothalamus, in relation to presentation of happy and sad pictures of strangers respectively, substantiating a more general role of the hypothalamus in social behavior beyond pair-bonded relationships.

Romantic relationships are posited to attenuate HPA axis-mediated stress-related activity [88] and to support regulation of neural responses to threat, pain, and social exclusion [61,65,71,89]. Coan and colleagues (2006), investigating the effects of social contact on threat responses, reported that women with higher-quality marital relationships had a significantly reduced activation of the hypothalamus when cued with a threat of an electric shock while holding their spouse’s hand; notably, hypothalamic activation positively correlated with higher ratings of unpleasantness of the electric shock. In a consecutive study, Brown and colleagues reported reduced hypothalamic activity in both men and women associated with partner’s handholding-induced decreased stress response, as well as with higher self-ratings of general health [89]. Similarly, López-Solà and colleagues (2019) observed that handholding of a romantic partner was significantly associated with hypoalgesia in women. The reduction of pain intensity and unpleasantness correlated with reduced activity in pain and stress-related core brain regions such as the hypothalamus and amygdala. Social contact-induced downregulation of hypothalamic activation has been observed not only in relation to physical pain reduction but it was also associated with mitigation of distress upon social exclusion [61]. Karremans and colleagues (2011) observed that the only reminder of an attachment figure attenuated the effects of social exclusion experienced by men and women taking part in a virtual ball-tossing game [67]. Notably, social exclusion attenuation by the psychological presence of the attachment figure was associated with reduced activity of the hypothalamus and other brain regions that were active while participants experienced social distress upon exclusion.

Altogether these results indicate that pair-bonding, and in particular a satisfactory romantic relationship, can moderate the effects of aversive events by directly modulating the activity of hypothalamus-mediated circuits underlying stress and defensive behavior.

The effects of attachment relationships are even evident in couples who have separated. Najib and colleagues observed decreased brain activity in the left hypothalamus along with other cortical and subcortical brain regions associated with grieving a romantic relationship breakup [60]. The authors reported these results in women whose romantic relationship ended within the preceding 4 months, with recalling sad and ruminative thoughts about their ex-partners as an indication of bond disruption and social separation [60].

On the other hand, Takahashi and colleagues showed differential brain activations in men and women in relation to jealousy-related behaviors [90]. Men but not women showed increased left hypothalamic activity during presentation of sentences depicting either emotional infidelity or sexual infidelity and no common activity was observed in both groups in typical socioemotional brain regions [90]. These results suggested higher distress in men in response to a threat of losing their bonded female partner, which, in particular cases, might also lead to dysfunctional aggressive behavior.

Finally, brain systems mediating pair bonding were also studied considering different cultures [62]. Xu and colleagues observed activations of reward and motivation systems when Chinese participants, involved in early-stage romantic love, were exposed to pictures of the face of their beloved [62]. Notably, the left hypothalamus activity appeared to be slightly correlated with increased endorsement of traditional values, possibly denoting a cultural influence on the affective experiential state.

### 3.3. Other Prosocial Interactions

In line with a more general role of the hypothalamus in mediating social interactions independently of pair and kinship bonds, additional studies reported involvement of the hypothalamus with respect to cooperative human behavior and social attitudes. Similar to previous evidence of the effects of attachment figures’ social contacts on mitigating pain and social distress [61,65,71,89], some studies observed that unfamiliar but supportive individuals can also lead to diminished hypothalamic-mediated stress responses. For instance, Eisenberger and colleagues demonstrated that social interaction with unknown but sympathetic individuals attenuates neuroendocrine-mediated social distress [91]. Encouraging interaction during a virtual ball-tossing game also diminished neural and physiological reactivity to a social stressor as evidenced by reduced hypothalamic response and cortisol reactivity [67]. In this study, further analysis of neural and endocrine reactivity revealed differential activations of hypothalamic subregions either correlating positively with cortisol response or negatively with social support, but no concurrent correlation with either cortisol reactivity or social support was observed. However, hypothalamic activation correlating negatively with social support showed a positive correlation with social distress, suggesting that some portions of the hypothalamus might represent a mediator between increased social interaction and reduced social distress [91]. In addition, the authors reported that the hypothalamic area associated with cortisol responses also correlated with dorsal ACC and Brodmann area 8, suggesting that activity of these two regions might modulate cortisol reactivity.

Several neuroimaging studies also reported hypothalamic engagement during reciprocal and cooperative behavior. In 2006, Moll and colleagues reported greater activity of the subgenual area and the ventral striatum together with the adjacent septo-hypothalamic region in participants willing to donate to real charitable organizations with respect to participants taking decisions leading to pure monetary rewards, notwithstanding a comparable involvement of the mesolimbic reward system [66]. Krueger and colleagues reported activation of the septo-hypothalamic region when participants, involved in a sequential reciprocal trust game with a stranger, made decisions of continuing the game, which resulted in a cumulative and equal monetary payoff for both, and hoping to receive a better payoff, instead of quitting the game, which resulted in a larger payoff for the defector and a payoff of zero for the partner [92]. Notably, by dividing the experiment into two stages—the partnership-building stage and partnership stage—the authors observed activation of the septo-hypothalamic area associated with unconditional trust towards the unknown partners [92]. Unconditional trust, referring to a previous assumption that a game partner was trustworthy, was assumed on the basis of the first preceding supportive and cooperative performance. On the contrary, conditional trust was dependent on strategies adopted at the second stage, where the benefits of cooperating, risk of defection, and the future value of past decisions were anew contingently assessed.

Hypothalamic activation has been also linked to other interpersonal behaviors, such as admiration and compassion [68], two socioemotional categories strongly affecting intersubjectivity. For example, neuroimaging data acquired when participants were exposed to emotional narratives based on true stories, and explicitly requested to recall and empathize with depicted emotional experiences, that is admiration for virtue or for skill, and compassion for social/psychological pain or for physical pain, altogether revealed activations of the anterior insula, anterior cingulate, hypothalamus, midbrain and other regions involved in interoceptive representation and homeostatic regulation. In particular, admiration for virtue and compassion for social/psychological pain was associated with greater activation of the anterior cingulate, anterior insula, and hypothalamus, suggesting that these areas more specifically support emotional reactions to another person’s psychological state.

Neural correlates of human reciprocity have been further investigated by considering interpersonal gratitude to an anonymous partner, who by intentionally sharing pain stimulation lessens the experience of participant pain [70]. Such intentional interpersonal behavior leading to increased closeness and reciprocity—assessed as increased money transferring to the partner—was associated with activation of the septum/hypothalamus and other value-related brain structures such as the ventromedial prefrontal cortex and ventral tegmental area.

The hypothalamus has also been shown to mediate the decision to keep interacting with a social partner or to switch to another [64]. Participants’ social stay decisions, which were apparently biased by rewards determined by the intentional generosity of social partners, as compared with non-prosocial decisions, where rewards were determined by an unintentional algorithmic process, were associated with activity of the septo-hypothalamic area along with the ventromedial prefrontal cortex and caudate nucleus.

Aiming to capture dynamic brain activity during more ecological social interactions, Rauchbauer and colleagues performed brain scanning while participants were involved in a natural conversational task either with a human being or with an anthropomorphic robot [72]. Human–human interaction as compared with human–robot interactions activated a bilateral cortico-subcortical network including the temporo-parietal junction, hypothalamus, thalamus, hippocampus, amygdala, and caudate nucleus [72]. These results, in combination with those of a previous report from the same group observing increased hypothalamic paraventricular nucleus (PVN) activity during human–human interaction with respect to human–robot interaction, in neurotypical as compared with ASD individuals [93], pointed to hypothalamic activity as a potential neural marker of social motivation towards human beings.

Recently, Bortolini and colleagues further reported an association between the septo-hypothalamic area and interpersonal affiliative behavior. In particular, they observed activity in this region during anticipation of presentation of videoclips of positive social scenes, depicting, for instance, erotic and tenderness interactions involving unfamiliar individuals [73]. Septo-hypothalamic activity during videos presentation correlated with positive arousal of affiliative rewards stimuli and motivation for subsequent re-exposure to them, but not with the same scores related to other non-affiliative rewarding stimuli. Notably, this study indicated that while the nucleus accumbens generally responds to rewarding stimuli of various natures, the septo-hypothalamic region appears to be more relevant to processing of social rewarding stimuli.

Finally, in one of the first attempts to disentangle the role of distinct hypothalamic nuclei in relation to the nature of social relationships, Wolfe and colleagues examined the differential involvement of the supraoptic nucleus and paraventricular nucleus during presentation of faces depicting a same-sex sibling, best friend, celebrity, and unknown person to university students [69]. The supraoptic nucleus responded to all familiar individuals, but sibling faces versus unknown faces activated the right supraoptic nucleus, friend faces the left supraoptic nucleus, whereas celebrity faces the supraoptic nucleus bilaterally. The right paraventricular nucleus was activated only when sibling faces were compared to best friend faces and correlated with the difference in familiarity scores but not with those of emotionality, suggesting an effect related to familiarity, possibly associated with the frequency of social interactions rather than to emotional feelings.

### 3.4. Laughter

Laughter is an instinctive and unconsciously controlled vocalization typically observable in social contexts such as during conversation, social play, as well as induced by humor [40,94]. Tickling-induced laughter is a quite old and conserved form of social physicality and communication allowing preverbal interaction between mother and infant and nonverbal communication between family, peers, and sexual partners. Tickling has been shown to induce positive affective responses [95] and to be significantly mediated by hypothalamic activity [41,96]. In healthy individuals, ticklish laughter induced by skin stimulation executed by a partner or a friend was associated with bilateral activation of the lateral hypothalamus along with other limbic areas such as the substantia nigra, amygdala, hippocampus, anterior insula, anterior cingulate gyrus, periaqueductal gray, and ventral tegmental area [41,96]. Activity in the lateral hypothalamus, the nucleus accumbens, and the ventral tegmental area was also observed in relation to anticipation of tickling, suggesting that these regions might support anticipatory mechanisms promoting social physicality [41].

In other types of social interactions, such as those occurring at pubs or restaurants, human laughter can be elicited by perception of humor and jokes typically concerning familiar individuals, although humoristic scenarios can also involve unfamiliar persons and even animals mimicking human characteristics and behaviors. In these situations laughter is a form of vocal grooming [97,98], and humor-related laughter represents a social rewarding experience promoting human communication and interaction and strengthening social bonds [99,100].

Mobbs and colleagues reported engagement of the hypothalamic region while individuals perceived humor [39]. The authors observed activation in a mesolimbic network including the anterior thalamus, the ventral striatum/nucleus accumbens, the ventral tegmental area, the hypothalamus, and the amygdala when participants were presented with funny cartoons, depicting individuals in comic situations, in contrast to non-funny cartoons [39]. In a subsequent study, Karlsson and colleagues confirmed involvement of the hypothalamus during processing of humor in participants presented with scenes depicting humans and animals (mimicking human poses) in comic situations with respect to neutral pictures [74]. Bilateral hypothalamic activation was observed in relation to both high-arousal positive and negative emotional stimuli, both including representations of human-related scenarios implying either con-participation to enjoyable context or empathic responses to dramatic experiences [74]. This study did not specifically aim to assess the association between hypothalamic activity and social responses, and consequently the authors discussed the involvement of the hypothalamus mainly in relation to arousal-induced motor inhibition, possibly mediated by hypocretin neurons, and not in the context of socioemotional behavior.

### 3.5. Social Threat

Some of the first neuroimaging evidence of an association between the hypothalamus and human response to social threat was observed in a study exploring the neural substrate underlying perception of dynamic whole-body expressions of anger in healthy individuals [75]. Observation of realistic angry dynamic expressions towards observers as compared with static stimuli elicited left hypothalamic activity, temporal pole, as well as premotor and ventromedial prefrontal cortex, whereas amygdala activity was associated with both static and dynamic angry expressions. These results suggested that the hypothalamus independently of amygdala might support modulation of autonomic reactions associated with defensive behavior. Hermans and colleagues also reported hypothalamic activity in relation to social threat during a baseline session of an experimental protocol implying testosterone administration. Female participants viewing angry faces as compared with neutral faces showed enhanced bilateral hypothalamic activity [76]. Notably, such activity negatively correlated with cortisol level and positively with the testosterone–cortisol ratio indicating that the hypothalamic response to social threat was directly modulated by the overall neuroendocrine balance.

In addition, hypothalamic activation upon exposure to social threat appears independent of explicit attention to the stimuli interactions [77,78]. In fact, Sinke and colleagues observed engagement of the hypothalamus while male and female participants either overtly or covertly attended movies showing threatening interactions of an attacker male person towards a female one, and not when exposed to teasing interactions [77]. Amygdala activation was measured in both conditions but was higher during the threatening interaction condition. Although in this study participants were only witnessing a threatening interaction not involving them directly, amygdala and hypothalamic activity were interpreted as preparation of defensive reaction to a potential attack also towards the observer.

A similar attention-independent hypothalamic activation was also reported by Pichon and colleagues showing that covert attention to videos of fearful or angry expressions, signaling potential threat towards participants, prompted activity in a brain network including the right posterior medial hypothalamus, periaqueductal gray, and premotor cortex [78]. These activations in response to threat stimuli were observed while participants performed a stimuli-related emotional task, and also during an unrelated neutral task. The observation of task-dependent amygdala modulation, resulting in higher activity during the emotional task, indicated that the hypothalamus-dependent reactivity to threatening stimuli, underlying a reflexive defensive behavior, might be relatively independent of the amygdala.

## 4. Conclusions

Our systematic review revealed that the hypothalamus is involved in multifaceted aspects of human socioemotional behavior going beyond its well-established role in maternal care and extending to various prosocial relationships with both familiar and unfamiliar individuals with different levels of complexity and including potential defensive responses to social threat. Moreover, hypothalamus-mediated supportive social interactions, with both familiar and unfamiliar individuals, have been shown to mitigate physical pain perception as well as social distress.

Considering that hypothalamic nuclei appear to modulate both human socioemotional responses and stress reactions to physical and social stimuli, these results indicate a clear relationship between socioemotional and neuroendocrine brain systems. Hypothalamic-mediated neural interactions would then promote motivated social behaviors aiming to generally attenuate human distress and to promote well-being. According to this assumption, preservation of formed social relationships would be then indispensable. Accordingly, aggressive and defensive responses might indeed represent protective behaviors towards infants, romantic partners, and more generally towards human fellows. The few here-identified investigations showing hypothalamic activation associated with social threat and aggression mainly involved unfamiliar individuals [75,76,77,78], and likely indicate fear-related stress reactivity rather than protective behavior. However, it is conceivable that hypothalamic activity might also critically regulate protective and defensive responses towards persons we have bonded with.

In conclusion, the studies we examined showed that hypothalamus nuclei are an essential brain area mediating interpersonal relationships, and seemingly quite specific of human–human interactions [72,93]. Remarkably, the observed heterogeneity of socioemotional responses mediated by the hypothalamus suggests its extended functional role for species survival and preservation, ranging from exploratory and approaching behaviors promoting social interactions to aggressive and avoidance responses protecting and defending the established social bonds.

## Figures and Tables

**Table 1:** List of reviewed studies.

Year	Authors	Socioemotional Context	Sample Size	Sex	Main Stimuli Comparison	Hypothalamic Region	MNI Coordinates (Cluster Peak)
2002	Lorberbaum, et al. [52]	Mother-infant interaction	N = 10	M = 0, F = 10	Cry stimuli vs control noise	Hypothalamus	0, −6, −7 0, −10, −3
2004	Bartels & Zeki [53]	Romantic and maternal interactions	N = 20	M = 0, F = 20	Own childs photographs vs other childs photographs	L hypothalamus	−3, −12, −17
2008	Strathearn, et al. [54]	Mother-infant interaction	N = 28	M = 0, F = 28	Own baby’s face vs unknown baby face	Bilateral hypothalamus	3, −8, −7 −5, −8, −8
2008	Noriuchi, et al. [55]	Mother-infant interaction	N = 13	M = 0, F = 13	Video clips of own infant vs unknown infants in two situation: play vs separation	R hypothalamus	6, −8, −4 4, −10, −12
2009	Strathearn, et al. [56]	Mother-infant interaction	N = 30	M = 0, F = 30	Own infant vs unknown infant pictures	L hypothalamus	−3, 3, −18
2012	Moll, et al. [57]	Parent-child interaction	N = 30	M = 14, F = 16	Affiliative-positive and negative vs non affiliative positive and negative	L septal/preoptic–anterior hypothalamic area	−3, 2, −14
2014	Ho, et al. [58]	Mother-infant interaction	N = 14	M = 0, F = 14	Own infant vs unknown infants pictures	R septal-hypothalamic area	8, 0, −12
2018	Li, et al. [59]	Father-infant interaction	N = 39	M = 39, F = 0	own infant cry vs unknown infant cry	L hypothalamus	−2, −12, −18
2004	Najib, et al. [60]	Romantic interaction	N = 11	M = 0, F = 11	Recalling of sad vs neutral thoughts	Hypothalamus	0, 0, 1
2011	Karremans, et al. [61]	Romantic interaction	N = 15	M = 5, F = 10	Attachment figure’s name vs non-attachment figure’s name	L hypothalamus	−1, −1, −14
2011	Xu, et al. [62]	Romantic interaction	N = 18	M = 8, F = 10	Romantic partner VS familiar acquaintance photographs	L hypothalamus	−2, 0, −11
2012	Acevedo, et al. [63]	Romantic interaction	N = 17	M = 7, F = 10	Partner vs highly familiar acquaintance images; conjunction partner and close friend	Bilateral hypothalamus	−10, −2, −7 2, −4, −6 11, −1, −9
2017	Heijne, et al. [64]	Romantic interaction	N = 26	M = 13, F = 13	Social vs nonsocial “stay” decisions	L septo-hypothalamic region	−5, 7, 0
2019	Acevedo, et al. [63]	Romantic interaction	N = 18	M = 11, F = 7	Partner vs familiar face	R hypothalamus	9, 6, −9 9, 0, −9
2006	Coan, et al. [65]	Prosocial interaction	N =16	M = 0, F = 16	Husband’s hand-holding vs anonymous male experimenter’ hand-holding	R hypothalamus	1, −13, −6
2006	Moll, et al. [66]	Prosocial interaction	N = 19	M = 10, F = 9	Decisions to donate vs pure monetary reward	R septo-hypothalamic region	Not available
2007	Eisenberger, et al. [67]	Prosocial interaction	N = 32	M = 13, F = 19	Social exclusion vs inclusion during a virtual ball tossinggame	Bilateral hypothalamus	10, −4, −4 6, 0, −8 −10, 0, −12
2007	Krueger, et al. [66].	Prosocial interaction	N = 44	M = 22, F = 22	Trust during social reciprocal trust game vs control game	L septo-hypothalamic region	−4, 4,−3
2009	Immordino-Yang, et al. [68]	Prosocial interaction	N = 13	M = 7, F = 9	Emotional vs non emotional narratives - Admiration for virtue & Admiration for skill & Compassion for social pain & Compassion for physical pain vs control; admiration for virtue and compassion for social/psychological pain vs control	Bilateral hypothalamus	0, −5, 1 −3, −11, 7 −0, −8, 8 3, −8, 1 −3, 5, 5
2017	Wolfe, et al. [69].	Prosocial interaction	N = 20	M = 6, F = 14	Several contrasts among pictures of friend, sibling and celebrity vs unknown	L and R hypothalamic supraoptic and paraventricular nuclei	−6, 3, –16 8, 3, –16 8, 5, –14 −6, –1, –16 3, 1, –10
2017	Brown, et al. [58]	Prosocial interaction	N = 75	M = 41, F = 34	Partner handholding vs stranger handholding	R hypothalamus	2, −12, −11
2017	Yu, et al. [70].	Prosocial interaction	N = 27	M = 11, F = 16	Sharing vs non-sharing pain stimulation	L hypothalamus	−3, 2, −14
2019	Lòpez-Solà, et al. [71]	Prosocial interaction	N = 30	M = 0, F = 30	Partner hand-holding vs holding an inert rubber device	Hypothalamus	Not available
2019	Rauchbauer, et al. [72]	Prosocial interaction	N = 24	M = 7, F = 17	Human vs robot agent interaction	Bilateral hypothalamus	Not available
2021	Bortolini, et al. [73]	Prosocial interaction	N = 23	M = 9, F = 14	Videoclips of affiliative vs non affiliative scences with unfamiliar individuals	Bilateral septo-hypothalamic region	−4, −4, −10 −2, 7, −6 0, 7, −6
2003	Mobbs, et al. [39]	Humor	N = 16	M = 7, F = 9	Funny vs nonfunny cartoons	Bilateral hypothalamus	Not available
2010	Karlsson, et al. [74]	Humour	N = 20	M = 16, F = 4	Funny and sad vs neutral pictures	R hypothalamus	6, −8, −8 6, −8, −12
2019	Wattendorf, et al. [41].	Tickling	N = 31	M = 10, F = 21	Tickling of the foot by a friend/partner vs monotonous foot contact	L posterior lateral hypothalamus	−5, −13, −12
2008	Pichon, et al. [75]	Social threat	N = 16	M = 9, F = 7	Angry dynamic expressions vs statis stimuli	L hypothalamus	−6, 0, −14 −6, 0, −12
2008	Hermans, et al. [76]	Social threat	N = 12	M = 0, F = 12	Angry faces vs neutral faces	R hypothalamus	8, 0, −8 8, 0, 0
2010	Sinke, et al. [77]	Social threat	N = 14	M = 5, F = 9	Movies of threatening vs teasing interactions	R hypothalamus	4, −6, −8
2012	Pichon, et al. [78]	Social threat	N = 16	M = 8, F = 8	Videos of fearful or angry expressions vs neutral expressions	R dorsal hypothalamus	10, −8, −4

## References

[1] Reeves A.G., Plum F. (1969). Hyperphagia, rage, and dementia accompanying a ventromedial hypothalamic neoplasm. Arch. Neurol..

[2] Gorman D.G., Cummings J.L. (1992). Hypersexuality following septal injury. Arch. Neurol..

[3] Rosa M., Franzini A., Giannicola G., Messina G., Altamura A.C., Priori A. (2012). Hypothalamic oscillations in human pathological aggressiveness. Biol. Psychiatry.

[4] Bejjani B.P., Houeto J.L., Hariz M., Yelnik J., Mesnage V., Bonnet A.M., Pidoux B., Dormont D., Cornu P., Agid Y. (2002). Aggressive behavior induced by intraoperative stimulation in the triangle of Sano. Neurology.

[5] Giustina A., Braunstein G.D. (2016). Hypothalamic Syndromes. Endocrinology: Adult and Pediatric.

[6] Kunwar P.S., Zelikowsky M., Remedios R., Cai H., Yilmaz M., Meister M., Anderson D.J. (2015). Ventromedial hypothalamic neurons control a defensive emotion state. eLife.

[7] Silva B.A., Mattucci C., Krzywkowski P., Murana E., Illarionova A., Grinevich V., Canteras N.S., Ragozzino D., Gross C.T. (2013). Independent hypothalamic circuits for social and predator fear. Nat. Neurosci..

[8] Wang L., Chen I.Z., Lin D. (2015). Collateral pathways from the ventromedial hypothalamus mediate defensive behaviors. Neuron.

[9] Mangieri L.R., Jiang Z., Lu Y., Xu Y., Cassidy R.M., Justice N., Xu Y., Arenkiel B.R., Tong Q. (2019). Defensive Behaviors Driven by a Hypothalamic-Ventral Midbrain Circuit. eNeuro.

[10] Lin D., Boyle M.P., Dollar P., Lee H., Lein E.S., Perona P., Anderson D.J. (2011). Functional identification of an aggression locus in the mouse hypothalamus. Nature.

[11] Meyer-Lindenberg A., Domes G., Kirsch P., Heinrichs M. (2011). Oxytocin and vasopressin in the human brain: Social neuropeptides for translational medicine. Nat. Rev. Neurosci..

[12] Quattrocki E., Friston K. (2014). Autism, oxytocin and interoception. Neurosci. Biobehav. Rev..

[13] Hammock E., Veenstra-VanderWeele J., Yan Z., Kerr T.M., Morris M., Anderson G.M., Carter C.S., Cook E.H., Jacob S. (2012). Examining autism spectrum disorders by biomarkers: Example from the oxytocin and serotonin systems. J. Am. Acad. Child Adolesc. Psychiatry.

[14] Torres N., Martins D., Santos A.J., Prata D., Verissimo M. (2018). How do hypothalamic nonapeptides shape youth’s sociality? A systematic review on oxytocin, vasopressin and human socio-emotional development. Neurosci. Biobehav. Rev..

[15] Wang D., Xue S.W., Tan Z., Wang Y., Lian Z., Sun Y. (2019). Altered hypothalamic functional connectivity patterns in major depressive disorder. Neuroreport.

[16] Schindler S., Geyer S., Strauss M., Anwander A., Hegerl U., Turner R., Schonknecht P. (2012). Structural studies of the hypothalamus and its nuclei in mood disorders. Psychiatry Res..

[17] Caria A., Ciringione L., Falco S. (2020). Morphofunctional Alterations of the Hypothalamus and Social Behavior in Autism Spectrum Disorders. Brain Sci..

[18] Guastella A.J., Mitchell P.B., Dadds M.R. (2008). Oxytocin increases gaze to the eye region of human faces. Biol. Psychiatry.

[19] Rimmele U., Hediger K., Heinrichs M., Klaver P. (2009). Oxytocin makes a face in memory familiar. J. Neurosci..

[20] Savaskan E., Ehrhardt R., Schulz A., Walter M., Schachinger H. (2008). Post-learning intranasal oxytocin modulates human memory for facial identity. Psychoneuroendocrinology.

[21] Domes G., Heinrichs M., Michel A., Berger C., Herpertz S.C. (2007). Oxytocin improves “mind-reading” in humans. Biol. Psychiatry.

[22] Kosfeld M., Heinrichs M., Zak P.J., Fischbacher U., Fehr E. (2005). Oxytocin increases trust in humans. Nature.

[23] Bethlehem R.A., van Honk J., Auyeung B., Baron-Cohen S. (2013). Oxytocin, brain physiology, and functional connectivity: A review of intranasal oxytocin fMRI studies. Psychoneuroendocrinology.

[24] Gordon I., Vander Wyk B.C., Bennett R.H., Cordeaux C., Lucas M.V., Eilbott J.A., Zagoory-Sharon O., Leckman J.F., Feldman R., Pelphrey K.A. (2013). Oxytocin enhances brain function in children with autism. Proc. Natl. Acad. Sci. USA.

[25] Kirsch P., Esslinger C., Chen Q., Mier D., Lis S., Siddhanti S., Gruppe H., Mattay V.S., Gallhofer B., Meyer-Lindenberg A. (2005). Oxytocin modulates neural circuitry for social cognition and fear in humans. J. Neurosci..

[26] Riem M.M., van I.M.H., Tops M., Boksem M.A., Rombouts S.A., Bakermans-Kranenburg M.J. (2013). Oxytocin effects on complex brain networks are moderated by experiences of maternal love withdrawal. Eur. Neuropsychopharmacol..

[27] Wittfoth-Schardt D., Grunding J., Wittfoth M., Lanfermann H., Heinrichs M., Domes G., Buchheim A., Gundel H., Waller C. (2012). Oxytocin modulates neural reactivity to children’s faces as a function of social salience. Neuropsychopharmacology.

[28] Domes G., Heinrichs M., Glascher J., Buchel C., Braus D.F., Herpertz S.C. (2007). Oxytocin attenuates amygdala responses to emotional faces regardless of valence. Biol. Psychiatry.

[29] Ne’eman R., Perach-Barzilay N., Fischer-Shofty M., Atias A., Shamay-Tsoory S.G. (2016). Intranasal administration of oxytocin increases human aggressive behavior. Horm. Behav..

[30] Harari-Dahan O., Bernstein A. (2014). A general approach-avoidance hypothesis of oxytocin: Accounting for social and non-social effects of oxytocin. Neurosci. Biobehav. Rev..

[31] Mierop A., Mikolajczak M., Stahl C., Bena J., Luminet O., Lane A., Corneille O. (2020). How Can Intranasal Oxytocin Research Be Trusted? A Systematic Review of the Interactive Effects of Intranasal Oxytocin on Psychosocial Outcomes. Perspect. Psychol. Sci..

[32] Andari E., Richard N., Leboyer M., Sirigu A. (2016). Adaptive coding of the value of social cues with oxytocin, an fMRI study in autism spectrum disorder. Cortex.

[33] Aoki Y., Yahata N., Watanabe T., Takano Y., Kawakubo Y., Kuwabara H., Iwashiro N., Natsubori T., Inoue H., Suga M. (2014). Oxytocin improves behavioural and neural deficits in inferring others’ social emotions in autism. Brain.

[34] Aoki Y., Yamasue H. (2015). Reply: Does imitation act as an oxytocin nebulizer in autism spectrum disorder?. Brain.

[35] Watanabe T., Abe O., Kuwabara H., Yahata N., Takano Y., Iwashiro N., Natsubori T., Aoki Y., Takao H., Kawakubo Y. (2014). Mitigation of sociocommunicational deficits of autism through oxytocin-induced recovery of medial prefrontal activity: A randomized trial. JAMA Psychiatry.

[36] Watanabe T., Kuroda M., Kuwabara H., Aoki Y., Iwashiro N., Tatsunobu N., Takao H., Nippashi Y., Kawakubo Y., Kunimatsu A. (2015). Clinical and neural effects of six-week administration of oxytocin on core symptoms of autism. Brain.

[37] Kanat M., Spenthof I., Riedel A., van Elst L.T., Heinrichs M., Domes G. (2017). Restoring effects of oxytocin on the attentional preference for faces in autism. Transl. Psychiatry.

[38] Parker K.J., Oztan O., Libove R.A., Mohsin N., Karhson D.S., Sumiyoshi R.D., Summers J.E., Hinman K.E., Motonaga K.S., Phillips J.M. (2019). A randomized placebo-controlled pilot trial shows that intranasal vasopressin improves social deficits in children with autism. Sci. Transl. Med..

[39] Mobbs D., Greicius M.D., Abdel-Azim E., Menon V., Reiss A.L. (2003). Humor modulates the mesolimbic reward centers. Neuron.

[40] Provine R.R. (2004). Laughing, Tickling, and the Evolution of Speech and Self. Curr. Dir. Psychol. Sci..

[41] Wattendorf E., Westermann B., Fiedler K., Ritz S., Redmann A., Pfannmoller J., Lotze M., Celio M.R. (2019). Laughter is in the air: Involvement of key nodes of the emotional motor system in the anticipation of tickling. Soc. Cogn. Affect. Neurosci..

[42] Moher D., Liberati A., Tetzlaff J., Altman D.G., Group P. (2009). Preferred reporting items for systematic reviews and meta-analyses: The PRISMA statement. PLoS Med..

[43] Lenzi D., Trentini C., Tambelli R., Pantano P. (2015). Neural basis of attachment-caregiving systems interaction: Insights from neuroimaging studies. Front. Psychol..

[44] De Boer A., van Buel E.M., Ter Horst G.J. (2012). Love is more than just a kiss: A neurobiological perspective on love and affection. Neuroscience.

[45] Mercado E., Hibel L.C. (2017). I love you from the bottom of my hypothalamus: The role of stress physiology in romantic pair bond formation and maintenance. Soc. Pers. Psychol. Compass.

[46] Swain J.E. (2011). The human parental brain: In vivo neuroimaging. Prog. Neuro-Psychopharmacol. Biol. Psychiatry.

[47] Wild B., Rodden F.A., Grodd W., Ruch W. (2003). Neural correlates of laughter and humour. Brain.

[48] Mashek D., Aron A., Fisher H.E. (2000). Identifying, evoking, and measuring intense feelings of romantic love. Rep. Res. Soc. Psychol..

[49] Koscik T.R., Tranel D. (2011). The human amygdala is necessary for developing and expressing normal interpersonal trust. Neuropsychologia.

[50] Conner O.L., Siegle G.J., McFarland A.M., Silk J.S., Ladouceur C.D., Dahl R.E., Coan J.A., Ryan N.D. (2012). Mom-it helps when you’re right here! Attenuation of neural stress markers in anxious youths whose caregivers are present during fMRI. PLoS ONE.

[51] Xia M., Wang J., He Y. (2013). BrainNet Viewer: A network visualization tool for human brain connectomics. PLoS ONE.

[52] Lorberbaum J.P., Newman J.D., Horwitz A.R., Dubno J.R., Lydiard R.B., Hamner M.B., Bohning D.E., George M.S. (2002). A potential role for thalamocingulate circuitry in human maternal behavior. Biol. Psychiatry.

[53] Bartels A., Zeki S. (2004). The neural correlates of maternal and romantic love. Neuroimage.

[54] Strathearn L., Li J., Fonagy P., Montague P.R. (2008). What’s in a smile? Maternal brain responses to infant facial cues. Pediatrics.

[55] Noriuchi M., Kikuchi Y., Senoo A. (2008). The functional neuroanatomy of maternal love: Mother’s response to infant’s attachment behaviors. Biol. Psychiatry.

[56] Strathearn L., Fonagy P., Amico J., Montague P.R. (2009). Adult attachment predicts maternal brain and oxytocin response to infant cues. Neuropsychopharmacology.

[57] Moll J., Bado P., de Oliveira-Souza R., Bramati I.E., Lima D.O., Paiva F.F., Sato J.R., Tovar-Moll F., Zahn R. (2012). A neural signature of affiliative emotion in the human septohypothalamic area. J. Neurosci..

[58] Ho S.S., Konrath S., Brown S., Swain J.E. (2014). Empathy and stress related neural responses in maternal decision making. Front. Neurosci..

[59] Li T., Horta M., Mascaro J.S., Bijanki K., Arnal L.H., Adams M., Barr R.G., Rilling J.K. (2018). Explaining individual variation in paternal brain responses to infant cries. Physiol. Behav..

[60] Najib A., Lorberbaum J.P., Kose S., Bohning D.E., George M.S. (2004). Regional brain activity in women grieving a romantic relationship breakup. Am. J. Psychiatry.

[61] Karremans J.C., Heslenfeld D.J., van Dillen L.F., Van Lange P.A. (2011). Secure attachment partners attenuate neural responses to social exclusion: An fMRI investigation. Int. J. Psychophysiol..

[62] Xu X., Aron A., Brown L., Cao G., Feng T., Weng X. (2011). Reward and motivation systems: A brain mapping study of early-stage intense romantic love in Chinese participants. Hum. Brain Mapp..

[63] Acevedo B.P., Poulin M.J., Brown L.L. (2019). Beyond romance: Neural and genetic correlates of altruism in pair-bonds. Behav. Neurosci..

[64] Heijne A., Rossi F., Sanfey A.G. (2018). Why we stay with our social partners: Neural mechanisms of stay/leave decision-making. Soc. Neurosci..

[65] Coan J.A., Schaefer H.S., Davidson R.J. (2006). Lending a hand: Social regulation of the neural response to threat. Psychol. Sci..

[66] Moll J., Krueger F., Zahn R., Pardini M., de Oliveira-Souza R., Grafman J. (2006). Human fronto-mesolimbic networks guide decisions about charitable donation. Proc. Natl. Acad. Sci. USA.

[67] Eisenberger N.I., Lieberman M.D., Williams K.D. (2003). Does rejection hurt? An FMRI study of social exclusion. Science.

[68] Immordino-Yang M.H., McColl A., Damasio H., Damasio A. (2009). Neural correlates of admiration and compassion. Proc. Natl. Acad. Sci. USA.

[69] Wolfe F.H., Deruelle C., Chaminade T. (2018). Are friends really the family we choose? Local variations of hypothalamus activity when viewing personally known faces. Soc. Neurosci..

[70] Yu H., Cai Q., Shen B., Gao X., Zhou X. (2017). Neural substrates and social consequences of interpersonal gratitude: Intention matters. Emotion.

[71] Lopez-Sola M., Geuter S., Koban L., Coan J.A., Wager T.D. (2019). Brain mechanisms of social touch-induced analgesia in females. Pain.

[72] Rauchbauer B., Nazarian B., Bourhis M., Ochs M., Prevot L., Chaminade T. (2019). Brain activity during reciprocal social interaction investigated using conversational robots as control condition. Philos. Trans. R. Soc. Lond. B Biol. Sci..

[73] Bortolini T., Melo B., Basilio R., Fischer R., Zahn R., de Oliveira-Souza R., Knutson B., Moll J. (2021). Striatal and septo-hypothalamic responses to anticipation and outcome of affiliative rewards. Neuroimage.

[74] Karlsson K.A., Windischberger C., Gerstl F., Mayr W., Siegel J.M., Moser E. (2010). Modulation of hypothalamus and amygdalar activation levels with stimulus valence. Neuroimage.

[75] Pichon S., de Gelder B., Grezes J. (2008). Emotional modulation of visual and motor areas by dynamic body expressions of anger. Soc. Neurosci..

[76] Hermans E.J., Ramsey N.F., van Honk J. (2008). Exogenous testosterone enhances responsiveness to social threat in the neural circuitry of social aggression in humans. Biol. Psychiatry.

[77] Sinke C.B., Sorger B., Goebel R., de Gelder B. (2010). Tease or threat? Judging social interactions from bodily expressions. Neuroimage.

[78] Pichon S., de Gelder B., Grezes J. (2012). Threat prompts defensive brain responses independently of attentional control. Cereb. Cortex.

[79] George C., Kaplan N., Main M. (1985). Adult Attachment Interview.

[80] Singewald G.M., Rjabokon A., Singewald N., Ebner K. (2011). The modulatory role of the lateral septum on neuroendocrine and behavioral stress responses. Neuropsychopharmacology.

[81] Atzil S., Hendler T., Feldman R. (2011). Specifying the neurobiological basis of human attachment: Brain, hormones, and behavior in synchronous and intrusive mothers. Neuropsychopharmacology.

[82] Carter C.S. (1998). Neuroendocrine perspectives on social attachment and love. Psychoneuroendocrinology.

[83] Bartels A., Zeki S. (2000). The neural basis of romantic love. Neuroreport.

[84] Hazan C., Shaver P. (1987). Romantic love conceptualized as an attachment process. J. Pers. Soc. Psychol..

[85] Buffone A.E., Poulin M.J. (2014). Empathy, target distress, and neurohormone genes interact to predict aggression for others-even without provocation. Pers. Soc. Psychol. Bull..

[86] Rodrigues S.M., Saslow L.R., Garcia N., John O.P., Keltner D. (2009). Oxytocin receptor genetic variation relates to empathy and stress reactivity in humans. Proc. Natl. Acad. Sci. USA.

[87] Uzefovsky F., Shalev I., Israel S., Edelman S., Raz Y., Mankuta D., Knafo-Noam A., Ebstein R.P. (2015). Oxytocin receptor and vasopressin receptor 1a genes are respectively associated with emotional and cognitive empathy. Horm. Behav..

[88] De Vries A.C., Glasper E.R., Detillion C.E. (2003). Social modulation of stress responses. Physiol. Behav..

[89] Brown C.L., Beckes L., Allen J.P., Coan J.A. (2017). Subjective General Health and the Social Regulation of Hypothalamic Activity. Psychosom. Med..

[90] Takahashi H., Matsuura M., Yahata N., Koeda M., Suhara T., Okubo Y. (2006). Men and women show distinct brain activations during imagery of sexual and emotional infidelity. Neuroimage.

[91] Eisenberger N.I., Taylor S.E., Gable S.L., Hilmert C.J., Lieberman M.D. (2007). Neural pathways link social support to attenuated neuroendocrine stress responses. Neuroimage.

[92] Krueger F., McCabe K., Moll J., Kriegeskorte N., Zahn R., Strenziok M., Heinecke A., Grafman J. (2007). Neural correlates of trust. Proc. Natl. Acad. Sci. USA.

[93] Chaminade T., Da Fonseca D., Rosset D., Cheng G., Deruelle C. (2015). Atypical modulation of hypothalamic activity by social context in ASD. Res. Autism Spectr. Disord..

[94] Provine R.R. (2014). Funny science: Review: Ha! The science of when we laugh and why and the humor code: A global search for what makes things funny. Cerebrum.

[95] Panksepp J., Burgdorf J. (2003). “Laughing” rats and the evolutionary antecedents of human joy?. Physiol. Behav..

[96] Wattendorf E., Westermann B., Fiedler K., Kaza E., Lotze M., Celio M.R. (2013). Exploration of the neural correlates of ticklish laughter by functional magnetic resonance imaging. Cereb. Cortex.

[97] Dezecache G.D., Dunbar R.I.M. (2012). Sharing the joke: The size of natural laughter groups. Evol. Hum. Behav..

[98] Provine R.R. (2013). Laughing, grooming, and pub science. Trends Cogn. Sci..

[99] Provine R.R. (2001). Laughter: A Scientific Investigation.

[100] Martin R.A. (2007). The Psychology of Humor: An Integrative Approach.

